# Physical symptoms as psychiatric manifestations in medical spaces: A qualitative study

**DOI:** 10.3389/fpsyt.2022.1074424

**Published:** 2023-01-04

**Authors:** Georgia F. Spurrier, Kai Shulman, Sofia Dibich, Laelia Benoit, Kenneth Duckworth, Andrés Martin

**Affiliations:** ^1^Yale College, New Haven, CT, United States; ^2^Yale School of Medicine, New Haven, CT, United States; ^3^Child Study Center, Yale School of Medicine, New Haven, CT, United States; ^4^QUALab, Qualitative and Mixed Methods Lab, A Collaboration Between the Yale Child Study and the Centre de Recherche en Épidémiologie et Santé des Populations (CESP Inserm Université Paris Saclay Université Versailles Saint-Quentin), Paris, France; ^5^National Alliance on Mental Illness, Arlington, VA, United States

**Keywords:** mental health, physical symptoms, psychosomatic, medical care, stress, qualitative methods

## Abstract

**Background:**

Mental health and physical health issues frequently co-occur, but the impact of the psychological wellbeing on the body's physical functioning remains poorly understood within medical spaces. Individuals living with psychiatric diagnoses in particular are at an increased risk for developing chronic health issues and may be especially disadvantaged by healthcare systems which treat the mind and body as separate entities.

**Methods:**

We used secondary analysis (SA) to analyze 30 semi-structured interviews of individuals living with a serious mental illness or reflecting on a family member living with a serious mental illness. We deliberately sampled participants who reflected on salient experiences with co-occurring physical and mental health symptoms. All participants were associated with the National Alliance on Mental Illness (NAMI), the nation's largest grassroots mental health organization. We coded interviews using qualitative thematic analysis with an interpretative phenomenological framework centered on participants' subjective experiences.

**Results:**

Our analyses uncovered physical health challenges which often occur in individuals living with a psychiatric illness, emphasizing the bidirectionality of mental and physical symptoms. We identified three overarching domains: (i) *manifestations*, in which participants reflected on how their body responded physically to mental states; (ii) *medical management*, in which they discussed challenging experiences seeking medical treatment for physical symptoms while living with a mental health condition; and (iii) *disjointedness*, in which they reflected on challenges in navigating poorly coordinated mental and physical healthcare systems.

**Discussion:**

Participants and their medical providers struggled to incorporate mental wellbeing and its impact on physical health into overall healthcare. Given common experiences with misdiagnoses, difficulties navigating health care, and significantly delayed treatment, medical spaces may be able to improve patient experiences and satisfaction by accounting for psychological influences on health outcomes.

**Conclusion:**

Greater integration of physical and mental health care in medical spaces could improve health outcomes and reduce challenges for patients seeking treatment.

*There is no real difference between space, or internal place, and the corporeal substance contained in it. The only difference lies in the way in which we are accustomed to conceive of them*.

–*Descartes*

## Introduction

Mental illness can reliably increase the risk for the development of physical illness, which can in turn translate into decreased life expectancies (1). Indeed, the WHO has estimated that people with serious mental illness (SMI) have a 10–25-year reduction in life expectancy (2). Chronic health problems are common in patients with severe mental illnesses, partly related to reduced exercise, adverse effects of psychotropic medication use, and poor services and monitoring (3, 4). Effects of psychotropic medication, which range from poor dental health to sexual dysfunction, to difficulties with physical activity, may seriously complicate physical health outcomes in subjects with SMI (4). Unfortunately, the physical health of patients with SMI is frequently overlooked, resulting in a lack of recording, monitoring, and assessment for a population that is already vulnerable to poor health outcomes (4).

Delineating the causes of physical health issues in patients with SMI is complex and affected by a mix of factors, contributing to service barriers (4). Various biological, social, and psychological factors have the propensity to impact both mental and physical health symptoms. For example, medically unexplained symptoms (MUS) are overlapping and poorly understood conditions that often present as a congregation of somatic symptoms, a result of intersecting biological and psychological factors (5, 6). In a 2015 study, MUS were estimated to cost the United States $256 billion annually in medical costs (7). These symptoms frequently co-occur with psychiatric disorders and can be distressing and invalidate the patient's experience, given their being poorly understood medically (5, 8). Symptoms that cannot be explained medically often strain the general practitioner-patient relationship and reduce trust in the structure and function of health systems (5).

Symptoms that surface after exposure to traumatic events can be similarly opaque and be classified as MUS (6). Environmental stressors such as trauma, exposure to environmental toxins, or lack of control in the workplace can all impact physical health and contribute to these symptoms (6, 9–11). The surfacing of mental and physical symptoms in the wake of traumatic events may in part be understood through *embodiment*—environmental events triggering impairments in biological systems inherent to the physiologic response to stress, which can contribute to the embedding of negative patterns of thinking (12).

The significant overlap between mental and physical health is not unique to specific disorders, but applicable across health conditions (13). And yet, such an intersection presents challenges to the medical management of concurrent physical and mental symptoms with no clear etiology. These difficulties take several forms: First, there is a muddled cause-effect relationship that can contribute to medical uncertainty, wherein it is difficult to pinpoint whether emotional distress causes somatic symptoms or the reverse is true (14). Second, management of co-occurring physical illness and mental health disorders is often suboptimal, given that dealing with the complexity of both can be daunting (8). This may in part be attributed to division of labor within medical specialties, wherein medical professionals often operate homogeneously within their segment of the profession, thus reducing their own uncertainty but causing more subtle aspects of care to be overlooked or diverted to other specialists (15, 16). Third, diagnostic overshadowing is common in patients with SMI, wherein an individual's physical symptoms are treated solely as a reflection of their psychiatric diagnosis, resulting in barriers to proper care (17). Finally, because mental illness labels and the stigma associated with them commonly call into question the validity of symptoms, patients often report difficulty in getting routine health assessments and physical health issues being dismissed by care providers (17, 18).

There is a need to consider mental and physical health as bidirectional and integrated. The disconnect between psychiatric and medical systems creates a false dichotomy in which mind and body are conceptualized as separate entities (5). This represents a critical gap, as most healthcare providers lack simultaneous specialization or expertise in both areas (14, 19). Minimal collaboration between primary and mental health care presents a burden for patients who may have to repeat assessments unnecessarily, are misinformed, or unsure as to which type of professional to consult for care (19).

In this qualitative study we explore the contemporaneous manifestation of physical symptoms in patients with mental health conditions, and their subsequent experiences with medical care.

## Methods

### Design and data collection

For this qualitative study, we used secondary analysis (SA) of semi-structured interviews to gather participant information. Secondary analysis is a method of analyzing qualitative data which explores research questions using existing data, allowing novel ideas to pursued without necessitating additional data collection efforts (20). Additionally, semi-structured interviews are a well-established method for exploring opinions and perceptions of individuals surrounding sensitive topics, as well as for probing ideas from samples with varied backgrounds and histories (21). One of the authors (KD) conducted the interviews over several months as part of a larger project with the National Alliance on Mental Illness (NAMI), the world's largest grassroots mental health organization, dedicated to improving the lives of Americans living with mental illness (22). The author (KD) interviewed individuals who were involved with NAMI in some capacity, or at times, individuals who had been referred to the project because of their unique stories or experiences which reflect living with mental illness. Data collection began in the summer of 2021 and was completed in fall of the same year. Analysis of the interviews began in the fall as the final interviews were being conducted. Identification of the final themes and writing of the manuscript began in early 2022. The semi-structured interviews were geared toward discussing participants' experiences and perceptions of living with mental illness, and their narratives surrounding the recovery process. All interviews took place online and were transcribed verbatim. We de-identified all transcripts which were then uploaded into NVivo (ISR International, Melbourne), a qualitative analysis software used for computerized support and organization of qualitative analysis.

In order to ensure we maintained rigor in this qualitative study, we assessed the quality of our manuscript using the Consolidated criteria for reporting qualitative research (COREQ) (23). These criteria consist of 32-items which help researchers to assess three domains of their work: research team and reflexivity, study design, and reporting and analysis (23). COREQ is designed to be used for in-depth interviews and serves to promote research reporting that is both complete and transparent in order to improve rigor and credibility (23). For this qualitative study, we maintained research rigor by assessing our manuscript against the comprehensive COREQ checklist, ensuring that all 32-items had been adequately addressed by our reporting.

### Participants

All participants in our sample are living with a mental illness, or reflected on a person close to them living with one. Having both personal and familial reflections on mental illness experiences allowed us to paint a more holistic picture of living with mental illness. The total sample was composed of 118 participants, a small number of whom were interviewed jointly given their relationships (siblings, parent and child, or romantic partnership). We intentionally sampled 30 individuals for this qualitative study from the overall sample obtained for NAMI. Two authors (GS, KS) reviewed and coded interviews separately in order to generate initial themes and later sorted these, identifying those that demonstrated concurrent physical and psychiatric health challenges. Our sample represents 30 individuals who recounted salient experiences with overlapping physical and psychiatric symptomatology during interviews. Information power, a concept used to guide sufficient sample sizes for qualitative work, posits that fewer participants are needed to conduct quality qualitative research when these participants hold more quality information relevant to the study's purposes (24). Our narrow study aim, the specificity of experience and knowledge of our participants, and the high quality of dialogue collected *via* interviews, all contribute to the information power of our study (24). Our working sample included 19 females and 11 males; 24 self-identified as white, four as Black or African American, one as Asian American or Pacific Islander, and one as Latinx.

We obtained ethics approval from the Yale University Institutional Review Board (HIC # 2000031331. Participants provided written informed consent as part of the larger project under NAMI, but written consent for this secondary and de-identified study waived by the IRB. Moreover, any potentially identifying information has been excluded from the results.

### Data analysis

We coded interviews using the principles of thematic analysis, a method that can be used to reveal complex, textured, and rich descriptions of collected data (25). We followed the 6-step approach to inductive thematic analysis developed by Braun and Clark (26). In this effort, we used interpretative phenomenological analysis (IPA) as a theoretical approach centered on the subjective experiences of participants (27, 28). Active participation of both researcher and participant is essential for IPA, as the researchers' conceptions are required to make sense of and interpret the personal world the participant is describing in a reflexive process known as a double hermeneutic (29). The author (KD) who recruited participants and conducted interviews is board certified in both adult psychiatry and child and adolescent psychiatry and serves as the chief medical officer of NAMI. Two authors (GS, KS) reviewed and coded interviews independently toward generating themes. We selected quotes for analysis by reviewing identified themes which related to our area of interest in the data. Additionally, and to ensure all relevant experiences were taken into account for our analysis, we conducted a text-search within NVivo of all interviews for keywords which may likely have been used to describe salient experiences with co-occurring physical symptoms and mental health conditions (e.g., “body”). At several time points across the coding process, all authors met to discuss, review, and triangulate codes and themes toward a final codebook. This process resulted in theoretical sufficiency, wherein additional data would not generate new themes, but rather be subsumed within already defined themes (30).

## Results

We developed three overarching domains, each with underlying themes and subthemes (Table 1). The first domain, “Manifestations”, explores how physical functioning may be affected by mental health conditions and underlying psychological influences. The second, “Medical management”, discusses the experiences of individuals with concurrent mental and physical health issues, aiming to expose potential gaps in care. The final domain, “Disjointedness”, addresses the benefits of considering mind and body as intrinsically intertwined, and the implications of such a synthetic conceptualization on medical spaces and the patient care provided within them. The following three sections will trace the stories of several participants, each of whom has been provided with a pseudonym.

**Table 1:** Domains, themes, and representative quotes.

**Domain**	**Theme**	**Representative quote(s)**
Manifestations	The body can respond physically to mental states	I experienced just an overall like a sensation on my neck that was very hot and my body was experiencing this. I can't even describe what it was. I had weird pains in my arm.
		I wanted relief now because it was so freaking painful…physical pain, like when you're going in the throes of that, those meltdowns that I was in, there was a physical pain…it's like someone is reaching in and ripping out your soul, like that's what it felt like.
		My anxiety and my stress over my children cause me physical illness.
Medical management	Physical symptoms can provide a facile explanation	I had Graves' disease and they had to nuke my thyroid. I had a thyroid storm and I thought I was having a heart attack and they put me in the ambulance and they're like, “Well, give her a milligram of Ativan.”
	Medical providers can effectively incorporate mental wellbeing into treatment	I figured out that the back pain that I thought forever was fibromyalgia, is often just extreme tension in my body from having hyper vigilance from being an active person…I remember the first time my [physical] therapist suggested a grounding exercise and I was like, “Why the hell would I want to do that?” But it's important to be able to know what your body's telling you…it was very beneficial for me to be able to inhabit my body, you know?
Disjointedness	Separate mental and physical health treatment settings pose a challenge for all patients Mental illness can delegitimize the experience and treatment of physical illness	When he was in the psychiatric hospital…they were talking to him and he literally collapsed. And I mean, lost all physical functioning, including bladder and all that. So they knew that he had a seizure of some sort…So they rushed him by ambulance to another hospital, which was a medical hospital, not a psychiatric hospital…And they ran every test known to mankind…and they could find nothing physically wrong. I guess maybe by a process of elimination…they diagnosed him with pseudo seizure.

### Manifestations

All participants in our sample had either personal or familial experience with mental illness; many experienced concurrent disruptions in physical functioning. Often, participants reflected on physical symptoms with worry or confusion, unsure of the underlying cause. This section and the next follow the stories of several individuals, each assigned an alphabetically consecutive pseudonym. Andrew, who has struggled with his body's responses to anxiety since he was young; Beth, whose husband had frightening physical symptoms of panic attacks; and Chloe, who had episodic blindness while also battling severe psychiatric symptoms. The following two sections also include complementary quotations from other participants, as the experiences of our three index individuals were prominently reflected across the overall sample.

#### Physical conditions can be missed in the context of mental illness

The body can respond physically to mental states and mental symptoms can be manifested bodily. “Andrew”, who struggles with anxiety, noticed from a young age how his body responds to the world around him. In the fifth grade, he experienced what he now presumes to be his first anxiety attack, which for him was characterized by sensory overload and feelings of his classroom walls closing in. These challenges with sensory stimulation continued into high school as well:

My body has always reacted to my anxiety. I've gotten weird manifestations in my body.

His first experience with an anxiety attack was one of overwhelming terror. Later diagnosed with an anxiety disorder, Andrew went on to understand how his body reflects subjective tension and stress. Similarly, bodily manifestations could be frightening for participants, who were most often struggling with primarily mental, rather than physical health conditions. Such physical responses exemplify how mental wellbeing does not exist in a vacuum, and that conceptualizing it that way may only result in confusion for patients.

Not everyone, and perhaps not even most, have Andrew's insight. Some experiences highlight the overlap between physical and mental health disruptions that may lead to challenges when seeking treatment. “Beth,” for example, has a daughter who struggles with serious mental illness as well as a husband with what she described as somewhat “minor” challenges with depression. In discussing how she communicated the family's complex mental illness history to her daughter, Beth reflected on a confusing experience with her husband:

[W]e didn't realize at the time, [and] for several years, [that he] had had panic attacks. But every time he did, it was like he was having a heart attack and we'd go to the hospital because we thought it was his heart.

Though it was eventually discovered that Beth's husband was dealing with a predominantly psychiatric diagnosis, both Beth and the doctors had inferred a physical ailment as the culprit. More broadly, participants struggled to distinguish between physical and mental causes of different physical manifestations. Sometimes, these manifestations were complete mysteries—stumping doctor and patient alike.

Disruptions in health of unknown etiology may pose a particular challenge to the wellbeing and timely treatment of patients. “Chloe,” an Asian American woman in her early twenties, reflected on her diagnoses of bipolar and post-traumatic stress disorders, and on her practice of various self-care methods as a means of adapting to a lack of understanding by her healthcare providers. Beginning in her teenage years, Chloe was episodically and painfully blind:

I was diagnosed with glaucoma when I was 14. And I was told it was this inflammation of the eyes that would basically increase my eye pressure. And so, it popped my eyes blank, basically…

Chloe had a diagnosis of bipolar disorder by then, but the underlying cause of her visual condition remained elusive. Unlike Beth's husband's symptoms, the manifestation of Chloe's visual symptoms were rare within the medical spaces she was seen in. Clinicians must think creatively about the connection between physical and mental diagnoses in order to treat patients most effectively, keeping in mind that our understanding of the mind-body connection is still rudimentary. After explaining her condition, Chloe reflected back on the challenges she faced in accessing care: systemically, cross-culturally, and relating to the stigma associated with engaging clinically with her mental illness.

#### The body can respond physically to mental states

Certain bodily symptoms are well known to be linked to mental health. In “Dana's” words,

I was just overwhelmed a lot. I started to have some stomach problems, which were linked to stress, so…I went to GI doctors for years. I had really bad acid reflux and I had irritable bowel syndrome, which are two anxious-people conditions.

Irritable bowel syndrome (IBS) is a good example of a diagnosis that once posed a mysterious challenge to doctors. Functional Gastrointestinal Disorders such as IBS have since been identified as tightly correlated with psychological and mental functioning; physicians are now better equipped to apply an integrated understanding of both mind and body to treat patients more effectively (31). This participant was one of the few within the sample able to identify how her physical symptoms were inextricably linked to her mental ones of anxiety.

In some cases, participants recognized the lasting impact that untreated mental health conditions can have on the body. “Elaine” spoke of witnessing her mother develop breast cancer while struggling with substance addiction, which she considers masked the diagnosis of the malignancy:

When you're in the state of addiction, your physical health beyond kind of satiating that chemical dependence really is not top of mind. And so, I think it was a year or maybe two years of this developing cancer that she was completely unaware of…This is something that really has been replicated throughout rural areas, folks who have really unaddressed physical illnesses as a result of addiction and unchecked mental illness that really just, I think, snowballs into something that is really irreversible to a certain time.

Even as this participant reflected back on her first-hand experience, she also noted the impact of unchecked mental illness on her broader community. Mental illness can manifest through symptoms and disrupted functioning that mask underlying physical illness.

### Medical management

Participants often consulted medical spaces in the pursuit of healing and understanding their physical symptoms. However, their symptoms were often met with uncertainty or incorrect diagnoses and treatments, delaying healing and consuming valuable time and resources. This section will discuss the medical management of the symptoms of Andrew, Beth, and Chloe, as well as provide reflections from other participants on their experiences with treatment of concurrent mental and physical illness in medical spaces.

#### Physical symptoms can provide a facile explanation

Andrew reflected back on his childhood diagnosis of pleurisy, the dated term for an inflammation of the pleura, the thin covering of the lungs. The physician mistook Andrew's body's responses to anxiety for a physical condition, medicating him accordingly. In a medical space, it was not intuitive that his physical symptoms could have arisen from a primary psychiatric condition:

I don't remember what led up to it, but I know I was taking medication for pleurisy, the doctor thought I had pleurisy. I'm not sure what type, it was anti-inflammatory I think maybe.

Beth's journey through medical care with her husband was similar. Her husband suffered from what initially appeared to be from chest pain portending heart attacks, thus prompting them to frequent the hospital's emergency room. She reflected on their experiences seeking treatment in medical spaces:

[H]e had a heart murmur and they would run tests and they would do caths and send him home. And so, we didn't realize that all those were panic attacks or that there was anything wrong with him.

In light of the medical team's approach to her husband's symptoms, Beth remained unaware of what her husband was suffering from. This was as emotionally consuming for Beth and her husband as it was costly for the medical system and its limited resources. Interestingly, Beth stated that they were unsure anything was “wrong with” her husband—evidence of how entrenched mental health stigma can affect the perception of doctors and patients alike, as though what happens within the body is more legitimate or “real” than what happens in the mind.

Doctors used a variety of resources in an attempt to crack Chloe's case as well. Diagnosed with bipolar disorder, she experienced episodic blindness attributed to any number of elusive ophthalmologic diagnoses:

So, it turns out, actually, many years later, because I did hundreds of tests and went to all these specialists, and nobody could figure out what the heck was causing it. And it turned out that it's most likely psychosomatic, because, after I finally got into a good mental health regimen, started seeing a therapist and got my medication, that's also where the eye episodes [finally] stopped.

After consulting several medical doctors and enduring a wide range of medical testing, Chloe's symptoms, previously attributed to an early (mis)diagnosis of glaucoma, resolved once she found adequate treatment for her mental health conditions. During her interview, Chloe said that she has now been episode-free for over 3 years. However, she noted that physical healthcare was much better structured than mental healthcare, and that she struggled with understanding treatment options, finding specialists, and experiencing cultural stigma in mental but not in physical health spaces. Even though Chloe's physical healthcare was superior to that for her mental health, treatment could have been more efficient to navigate had the physical health spaces she visited placed greater importance on psychological influences on health from the outset.

#### Medical providers can effectively incorporate mental wellbeing into treatment

Andrew, Beth, and Chloe were misdiagnosed and overzealously tested without much initial consideration of their mental health. But some participants had a more integrated experience. “Frida” explained her visit to the doctor's office after struggling with strong emotional symptoms and losing her desire to eat:

Originally, they tested my thyroid because I come from a family that has I think hyperthyroidism… I went, they checked my thyroid levels. I believe they did blood work. They were looking at what I was eating and everything. And my doctor was kind of like, everything is coming back normal. Blood work is fine, your thyroid is fine. So once she kind of canceled out any physical elements that maybe would link to it, then she recommended and referred me to a psychiatrist.

After having several medical tests show normal results, Frida's primary care physician was able to refer her to a psychiatrist, where she was promptly diagnosed and treated for major depressive and generalized anxiety disorders. As in Chloe's case, it was after properly addressing her mental health that she finally began to see improvement in her symptoms. Because of her clinician's knowledge of psychological influences on health and their referral to a mental health specialist, Frida was able to readily access the treatment she needed.

In turn, “Gina” spoke about her mental health causing physical symptoms, which prompted her doctor to point out psychological and environmental influences potentially affecting her health:

I was getting anxiety in my throat, palpitations, I couldn't figure it out, my blood pressure was fluctuating. Then the good thing about it, what I liked about the doctor was that he was an African American male, and so we were able to talk about the issues of race in America… and so we were able to drill down to what's *really* going on and talk about my childhood, about how race played into my education at school, at my public school, how race played into the media and the news and my perception of myself, how race played into my workforce experience. And I was like damn, race made a big deal and contributed to my mental health.

It is critical to understand the far-reaching effects of the outside world on health symptomatology and wellbeing, mental as well as physical. While Gina's body was showing symptoms, her healing required a medical system informed by larger issues such as systemic racism. Additionally, her doctor's identity as an African American man allowed for a productive conversation regarding her experiences and how those formative and ongoing experiences would be likely to affect her health.

### Disjointedness

Participants in our sample struggled to receive treatment that considered and addressed their whole personhood. All too often, treatments were disconnected, treating the mind or the body but seldom both. Participants' mental health not only interfered with their physical wellbeing, but also affected the quality of their treatment. This section will discuss participants perceptions of and challenges with the medical system. In this third and final overarching domain we discuss how having a mental illness affects interpretations of physical illness, and the burdens placed on patients by having to navigate separate mental health and physical health systems.

#### Mental illness can delegitimize the experience and treatment of physical illness

Mental illness is highly stigmatized, including in medical spaces. This reality can affect the quality of care that patients with a diagnosed mental illness are able to receive, even when that care is related to physical wellbeing. Several participants reflected on the systemic barriers they encountered in medicine. “Henry” suggested how mental illness should be responded to and proposed an idea regarding why mental illness diagnosis may create barriers to care:

My first thing is to say, “Tell me how you would respond if a person has suffered from liver disease, or heart disease, or any other illness, how would you react? It should be no different than the person with mental illness.” Now, the issue is folks can't *see* the manifestation of mental illness, so it's somewhat of a surprise to them when one has an episode. How they cope with that, how do they keep the person safe, what they may do is difficult because the system is broken.

Henry, along with several others, conceptualized mental illness as a brain disease that should not be treated differently from any other organic illness. He noted that one of the primary reasons for a mental illness diagnosis to elicit a subpar treatment is because mental illness may be less noticeable to the eye, and by extension be interpreted as less real. He also echoes a sentiment held by many participants in the broader sample: the system is broken and needs change.

Discussing providing a mental health training for nursing students, “Iris” talked about how a diagnosis can affect treatment in medicine:

Because I had had experiences in hospitals where as soon as they hear there's a mental health condition, they disregard anything physical you have come to the hospital for… I think the biggest thing for them was that [the training] humanized [mental health] for them because so many people are just a name on a medical file and that humanized what mental health was all about.

Iris, previously diagnosed with bipolar disorder herself, initially reflected back on her own physical health being overlooked in hospitals. Like most other participants, she is using her own challenges navigating the health system to motivate improvements that might benefit others. She viewed improved treatment of psychiatric patients—herself included—as a way to move toward humanization and destigmatization in medicine.

#### Separate mental and physical health treatment settings pose a challenge for all patients

Physical and mental wellbeing are deeply integrated, and treatment spaces that consider physical or mental health in isolation may not be treating the whole patient. Patients would likely benefit from more interdisciplinary thinking and greater consideration of the overlaps between physical and mental health. “Jenna” commented on why she feels practitioners are unable to more deeply address mental influences on health:

Physicians don't have the time to cover all of the stuff that they could do with mental health. With them they're trying to check to make sure that you're still healthy with physical stuff and bodily things and stuff like that. They don't have enough time to cover both subjects.

Jenna expressed her concern in the context of a legislative change she was in the process of advocating for in her home state—making insurance cover not just physical wellness visits, but those for mental wellness as well. Jenna's passions for mental health advocacy stem from the high rates of suicide at her high school which she was privy to. Her comment reflects a more systemic issue, the current lack of time, resources, or will to assess mental wellbeing within general practice. Unfortunately, general practice is where patients often consult practitioners regarding physical symptoms, among them those that arise from, or are modified by problems in their mental health.

“Katja” talked about seeking treatment for her son, diagnosed with schizoaffective disorder. She encountered significant challenges after having to consult various medical specialists, all of whom failed to treat him as a whole person:

That is actually why we started flying to New York, because none of the psychiatrists would treat the whole body. They only want to prescribe an antipsychotic. Then you go to your family doctor and they say, “I'm sorry, you need to go back to your psychiatrist.” …And our family doctor would not give him blood pressure medicine. He kept saying it was part of his psychosis. So all the other doctors, besides the psychiatrist, want to blame everything on the mental illness and not treat it. So you have all of these patients that have bipolar or schizophrenia or whatever, and they're only getting the anti-psychotic or the antidepressant and everything else is neglected.

Katja struggled accessing necessary treatment for her son in both psychiatric and general family medical care due to a lack of understanding and collaboration across specialties. She and her son had to travel for treatment, fight to receive necessary medication, and experience denial of his symptomatology because of his mental illness. A lack of understanding regarding how mental health affects physical health in medical spaces can create a tiring and burdensome path to recovery for patients. Participants' reflections in this final domain reveal the gap in incorporating into care the deep integration and overlap of mental and physical wellbeing.

## Discussion

By analyzing interviews of patients living with psychiatric diagnoses, we uncovered physical health challenges that frequently occur in this group. We identified three key domains: manifestations, medical management, and disjointedness. By following the stories of three participants and interweaving the experiences of several others, we underscore how psychiatric symptoms can manifest physically, resulting in medical mismanagement of underlying conditions. These results highlight the bidirectional nature of physical and mental wellbeing and generate implications for a more seamlessly enmeshed, collaborative approach to treatment in medical spaces.

Environmental and societal factors may have contributed to the overlap in participants' mental and physical health symptoms. The co-occurrence of mental and physical health disruptions may be impacted by the socio-economic costs of living with a mental health disorder, environmental stressors that affect both the brain and the body, including psychiatric medication side effect burden, which can cause deleterious metabolic outcomes (4, 14). Adverse events in childhood have been shown to pose a lifelong burden on physical health and subsequent quality of life, revealing the intersection between mental and physical health across the lifespan (9). Violence exposure has also been proven to cause lasting changes in neuroendocrine and central nervous system function, which can in turn contribute to a range of medically unexplained conditions and chronic physical symptoms (10, 11). Additionally, a recent meta-analysis identified growing evidence linking experiences with discrimination to greater distress and depressive symptoms (32). Racial discrimination is associated with poorer physical health outcomes across several axes, with notable increases in cardiovascular disease, incidence of obesity, and markers of inflammation, among others (32, 33). Experiencing racism also increases odds of low trust in health care systems and reduces satisfaction in the patient-provider relationship (32). Cultural conceptualizations of mental illness, which are particularly influential from the West, can also affect not only prioritization of symptoms and course of healing, but also the illness itself (34).

Interwoven in several of our findings were experiences of stigmatization as a result of mental health status. It has been suggested that stigma in itself may strongly contribute to the physical health disparities seen in individuals with mental illnesses (1). As such, stigmatization may be another factor contributing to the prevalence of co-occurring mental and physical health conditions. To tackle these physical health disparities in patients with mental illness, several prior pieces of literature have suggested more integrated and collaborative health care (4, 5, 14, 17, 35). In many cases, participants reverted to physical explanations of their symptoms, which may have been driven by a desire to be protected from mental health stigma or to protect a sense of self (5). Challenges to navigate the healthcare system and to receive proper care as a consequence of a mental health diagnosis may induce patients to lose faith in the healthcare system and opt to avoid it. Reducing the dualistic nature of physical and mental health care has the potential to ease the treatment-seeking process for those with coincident mental health and physical health symptomatology. Findings from our sample evidence current obstacles for patients with concurrent challenges because of a dualistic and balkanized healthcare system.

After the onset of challenges with their physical health, our participants often showed feelings of confusion and frustration surrounding their symptoms' origin, which were not well mitigated by consulting medical spaces. Participants in other samples have also expressed the burdensome nature of repeated assessments due to the lack of collaboration between providers, akin to the experiences of some individuals in our sample (19). Our results primarily reflect the perspectives of patients rather than of medical providers, but providers' similar confusion in response to our participants' physical symptoms was also apparent. Diagnoses that overlap psychiatric and general medicine are muddled and may have affected the diagnostic errors experienced by some of our participants. Due to the lack of clarity surrounding which provider is responsible for treating what overlapping symptom constellation, persons with mental health disorders may have their physical health overlooked and under-monitored (4).

Greater collaboration across health specialties could improve the integration of psychological influences on health into medical care—though this may require pushing back on traditional structures and roles (5). For example, it may be necessary to push back on division of labor within medical specialties, thus encouraging medical professionals to operate in novel segments of the profession, embrace uncertainty, and adopt new skills in order to appreciate the complexity of diagnosis and treatment (15, 16). There is evidence of psychological therapy resulting in improvements in syndromes that seemed untreatable by general medicine alone. MUS are often treated using cognitive behavioral therapy (CBT) or Dynamic Interpersonal Therapy (DIT), which recognize the emotional and relational components of these symptoms (5). Being knowledgeable about one's diagnosis can create a sense of control and hope, and is a process that may be improved through the integration of psychological principles (36). The absence of coordinated and well-integrated physical and mental health care represents a critical gap to optimal treatment and services for this population (35).

The practice of clinical general medicine and psychiatry could both improve their treatment outcomes by developing education initiatives addressing barriers to care identified by our participants. These include stigmatization, discrimination, and lack of knowledge regarding the interconnectedness of mind and body. Medical spaces should have adequate capacity to allow patients with mental illness to access necessary prevention, screening, and treatment of their physical health (35). Future research should examine the perspective of persons working in different areas of care and administration in medical spaces. Investigating barriers to proper care by integrating the perspectives of patients such as those in this study, and by medical providers from future work, could allow for a more nuanced examination of, and reduction in barriers to optimal physical healthcare for individuals with mental illness.

## Limitations

Our study has several noteworthy limitations. First, a review of the literature shows that patients may be resistant to psychological explanations of their physical symptoms. Medically-based explanations are often more convenient and desirable to patient and physician alike, which may limit the generalizability of our findings to patients without a preexisting psychiatric diagnosis (6). Other qualitative work has shown that psychological illness explanations can feel dismissive and stigmatizing and leave individuals feeling ashamed or disbelieved (5, 13).

Second, our study pulled from semi-structured interviews on mental illness and recovery broadly defined (given that data came from interviews for a separate project), rather than on specifically inquired experiences with concurrent physical health issues. Given this focus, the stories shared by participants of their physical health and medical care challenges may only represent highly salient memories, and many important challenges may not have been picked up by these interviews. However, participants' reflecting on their health challenges despite this not being the primary focus of interviews, reiterates the salience of these experiences for individuals living with mental illness.

Finally, our interviews did not include the complementary views of providers, which are critically important for designing feasible approaches to improving care in medical spaces.

## Conclusion

By using thematic analysis of thirty semi-structured interviews, we uncovered salient experiences of medically unexplained symptoms and physical illness in a sample of participants reflecting on their mental illnesses. We divided reflections into three domains: *manifestations* of psychiatric illness as physical symptoms; *medical management* of those coincident symptoms; and the *disjointed* nature of medical spaces catering separately to physical and mental health needs. Our findings emphasize the significant role of mental wellbeing in patient-centered care and optimal physical health outcomes—a role that is poorly understood by both patients and medical providers. Expanding training and education opportunities regarding psychological influences on health, and promoting a more seamless collaboration between medicine and psychiatry may enhance proper treatment for patients with overlapping health issues.

## Data availability statement

The raw data supporting the conclusions of this article will be made available by the authors, without undue reservation.

## Ethics statement

The studies involving human participants were reviewed and approved by the Yale University Institutional Review Board (HIC # 2000031331). Written informed consent to participate in this study was provided by the participants' legal guardian/next of kin. Written informed consent was obtained from the individual(s), and minor(s)' legal guardian/next of kin, for the publication of any potentially identifiable images or data included in this article.

## Author contributions

GS was a major contributor in data analysis and writing (drafting and editing). LB and AM contributed substantially to conceptualization, study design, and writing (review and editing). KS and SD contributed to data analysis, conceptualization, and writing (editing). KD was a major contributor to conceptualization, interview of subjects, study design, and data curation. All authors read and approved the final manuscript.
